# *N*-(2-Benzoyl­eth­yl)propan-2-aminium chloride

**DOI:** 10.1107/S1600536812035106

**Published:** 2012-08-11

**Authors:** Abdullah Aydın, Mehmet Akkurt, Halise Inci Gul, Ebru Mete, Ertan Sahin

**Affiliations:** a Department of Science Education, Faculty of Education, Kastamonu University, 37200 Kastamonu, Turkey; b Department of Physics, Faculty of Sciences, Erciyes University, 38039 Kayseri, Turkey; c Department of Pharmaceutical Chemistry, Faculty of Pharmacy, Atatürk University, 25240 Erzurum, Turkey; d Department of Chemistry, Faculty of Sciences, Atatürk University, 25240 Erzurum, Turkey

## Abstract

In the title salt, C_12_H_18_NO^+^·Cl^−^, N—H⋯Cl inter­actions between the free chloride anions and the organic cations connect the mol­ecules into hydrogen-bond dimers, forming a *R*_2_^2^(8) motif. The dimers are linked by C—H⋯O hydrogen bonds into chains extending along [301]. The carbonyl group is co-planar with the phenyl ring [C—C—C=O torsion angle = −3.3 (7)°]. The side chain has an *E* conformation.

## Related literature

For the details of the pharmacological effects of Mannich bases and for their synthesis, see: Dimmock & Kumar (1997 ▶); Gul *et al.* (2004 ▶, 2005*a* ▶,*b* ▶, 2009 ▶); Mete *et al.* (2011*a* ▶,*b* ▶); Kucuk­oglu *et al.* (2011 ▶); Canturk *et al.* (2008 ▶); Chen *et al.* (1991 ▶); Gul (2005 ▶); Suleyman *et al.* (2007 ▶); Plati *et al.* (1949 ▶). For bond-length data, see: Allen *et al.* (1987 ▶). For hydrogen-bond motifs, see: Bernstein *et al.* (1995 ▶); Etter (1990 ▶). For some related structures, see: Abonia *et al.* (2011 ▶); Tuzina *et al.* (2006 ▶). 
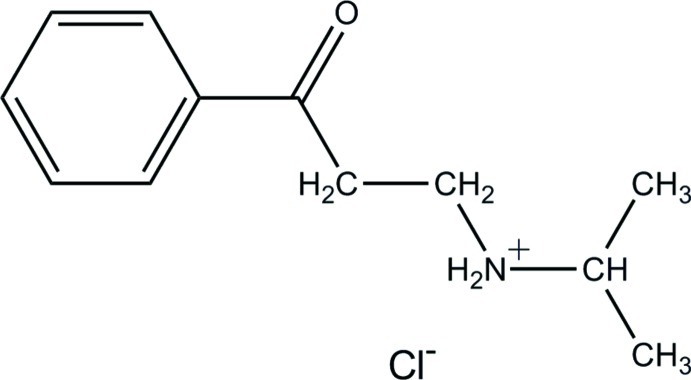


## Experimental

### 

#### Crystal data

C_12_H_18_NO^+^·Cl^−^
*M* *_r_* = 227.72
Monoclinic, 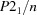

*a* = 8.036 (5) Å
*b* = 8.656 (5) Å
*c* = 18.403 (5) Å
β = 97.174 (5)°
*V* = 1270.1 (11) Å^3^
*Z* = 4
Mo *K*α radiation
μ = 0.28 mm^−1^
*T* = 294 K
0.16 × 0.13 × 0.12 mm

#### Data collection

Rigaku R-AXIS RAPID-S diffractometer
Absorption correction: multi-scan (Blessing, 1995 ▶) *T*_min_ = 0.958, *T*_max_ = 0.967
15799 measured reflections
2334 independent reflections
1204 reflections with *I* > 2σ(*I*)
*R*_int_ = 0.116

#### Refinement

*R*[*F* ^2^ > 2σ(*F* ^2^)] = 0.075
*wR*(*F* ^2^) = 0.224
*S* = 1.05
2334 reflections
145 parameters
2 restraints
H atoms treated by a mixture of independent and constrained refinement
Δρ_max_ = 0.49 e Å^−3^
Δρ_min_ = −0.19 e Å^−3^

### 

Data collection: *CrystalClear* (Rigaku/MSC, 2005 ▶); cell refinement: *CrystalClear*; data reduction: *CrystalClear*; program(s) used to solve structure: *SHELXS97* (Sheldrick, 2008 ▶); program(s) used to refine structure: *SHELXL97* (Sheldrick, 2008 ▶); molecular graphics: *ORTEP-3 for Windows* (Farrugia, 1997 ▶) and *PLATON* (Spek, 2009 ▶); software used to prepare material for publication: *WinGX* (Farrugia, 1999 ▶).

## Supplementary Material

Crystal structure: contains datablock(s) global, I. DOI: 10.1107/S1600536812035106/qm2079sup1.cif

Structure factors: contains datablock(s) I. DOI: 10.1107/S1600536812035106/qm2079Isup2.hkl

Supplementary material file. DOI: 10.1107/S1600536812035106/qm2079Isup3.cml

Additional supplementary materials:  crystallographic information; 3D view; checkCIF report

## Figures and Tables

**Table 1:** Hydrogen-bond geometry (Å, °)

*D*—H⋯*A*	*D*—H	H⋯*A*	*D*⋯*A*	*D*—H⋯*A*
N1—H1*N*⋯Cl1^i^	0.87 (4)	2.30 (4)	3.141 (4)	163 (4)
N1—H2*N*⋯Cl1	0.87 (3)	2.27 (3)	3.131 (4)	169 (4)
C10—H10⋯O1^ii^	0.98	2.56	3.284 (7)	130

Symmetry codes: (i) 
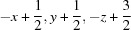
; (ii) 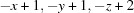.

**Fig. 1.**
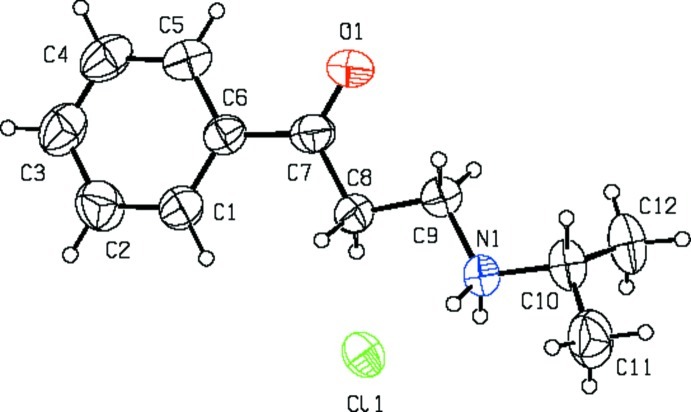
View of the title molecule with the atom numbering scheme. Displacement ellipsoids for non-H atoms are drawn at the 30% probability level.

**Fig. 2.**
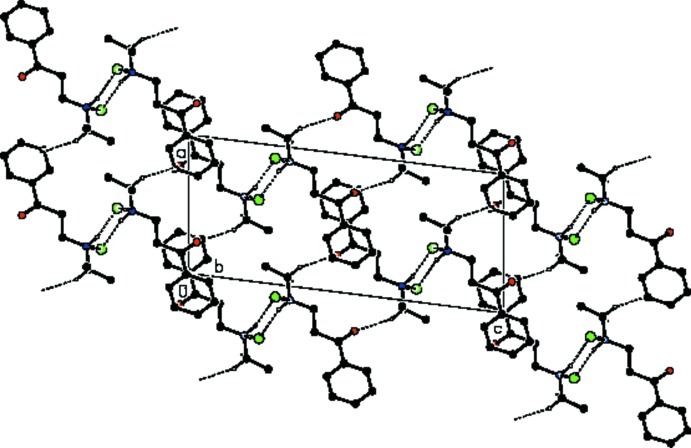
The packing and hydrogen bonding of (I) viewed down the b axis. H atoms not involved in hydrogen bondings are omitted for the sake of clarity.

## supplementary crystallographic information

### Comment

Mannich bases are generally formed by the reaction between formaldehyde, a secondary amine and a compound containing reactive hydrogen atoms. On occasion, aldehydes other than formaldehyde may be employed and the secondary amine may be replaced by ammonia and primary amines. This process is known as the Mannich reaction (Dimmock & Kumar, 1997).

Mannich bases display varied biological activities such as antimicrobial (Gul *et al.*, 2005; Mete *et al.*, 2011*a*), cytotoxic (Gul *et al.*, 2005; Mete *et al.*, 2011*b*; Kucukoglu *et al.*, 2011; Canturk *et al.*, 2008), anticancer (Dimmock & Kumar, 1997; Chen *et al.*, 1991; Gul, 2005), antiinflammatory (Suleyman *et al.*, 2007; Gul *et al.*, 2009), anticonvulsant (Gul *et al.*, 2004) and DNA topoisomeraseI inhibiting properties (Canturk *et al.*, 2008).

The geometric parameters of the title salt (I) in Fig. 1 are within the range of expected values for this type of compound (Allen *et al.*,1987; Abonia *et al.*, 2011; Tuzina *et al.*, 2006).

The N—H···Cl hydrogen-bonding interactions between the free chloride anion and the organic cation link the molecules into hydrogen-bond dimers, forming an *R*^2^_2_(8) motif (Bernstein *et al.*, 1995; Etter, 1990). The dimers are linked by C—H···O hydrogen bonds, into chains extended along the [301] direction (Table 1, Fig. 2).

### Experimental

A mixture of the appropriate ketone (50 mmol), paraformaldehyde (50 mmol), and isopropylamine hydrochloride (27 mmol) was heated in an oil bath at 403 K. The reaction vessel was then removed from the oil bath and when the temperature of the mixture dropped to 338 K, ethyl acetate (40–80 ml) was added. The mixture was stirred at room temperature for 24 h and the resultant precipitate was collected and recrystallized from ether/methanol. The melting point was 445–447 K (lit. Plati *et al.*, 1949 m.p. 447–449 K) and the yield was 55% (Mete *et al.*, 2011*b*).

^1^H-NMR δ 1.49 (d, J = 6.6 Hz, 6H, CH(CH_3_)_2_), 3.36–3.42 (m, 3H, CH(CH_3_)_2_ and 2 *x* H-2), 3.77 (t, J = 7.5 Hz, 2H, 2 *x* H-3), 7.36 (t, J = 7.3 Hz, 2H, H-3'/5'), 7.51 (t, J = 7.3 Hz, 1H, H-4'), 7.90 (d, J = 7.3 Hz, 2H, H-2'/6'), 9.56 (brs, 2H, NH_2_^+^); ^13^C-NMR δ 19.4 (CH(CH_3_)_2_), 35.2, 40.3, 51.2, 128.3, 128.9, 134.0, 136.0, 196.9; MS (EI) m/*z* (%): 176.2 (M–CH_3_)^+^, 192.1 (*M*+H)^+^. IR (KBr, cm-1): 2453 (NH_2_^+^), 1678 (CO). Calcd for C_12_H_18_ClNO (227.73): C, 63.29; H, 7.97; N, 6.15. Found: C, 63.26; H, 8.18; N, 6.23.

### Refinement

H atoms of the NH_2_ group were located in a difference Fourier map. Their positions refined with restraints on the N—H bond lengths of 0.86 (2) Å, while their thermal parameters were refined as riding with *U*_iso_(H) = 1.2*U*_eq_(N). H atoms bound to C atoms were positioned geometrically, with C—H = 0.93(aromatic), 0.97(methylene) and 0.98 Å (methine), and refined as riding with *U*_iso_(H) = 1.5*U*_eq_(O) for methyl H and 1.2*U*_eq_(C) for the others.

### Crystal data

**Table:**

C_12_H_18_NO^+^·Cl^−^	*F*(000) = 488
*M**_r_* = 227.72	*D*_x_ = 1.191 Mg m^−^^3^
Monoclinic, *P*2_1_/*n*	Mo *K*α radiation, λ = 0.71073 Å
Hall symbol: -P 2yn	Cell parameters from 3090 reflections
*a* = 8.036 (5) Å	θ = 2.2–26.4°
*b* = 8.656 (5) Å	µ = 0.28 mm^−^^1^
*c* = 18.403 (5) Å	*T* = 294 K
β = 97.174 (5)°	Block, white
*V* = 1270.1 (11) Å^3^	0.16 × 0.13 × 0.12 mm
*Z* = 4	

### Data collection

**Table:**

Rigaku R-AXIS RAPID-S diffractometer	2334 independent reflections
Radiation source: Sealed Tube	1204 reflections with *I* > 2σ(*I*)
Graphite Monochromator monochromator	*R*_int_ = 0.116
Detector resolution: 10.0000 pixels mm^-1^	θ_max_ = 25.5°, θ_min_ = 2.2°
dtprofit.ref scans	*h* = −9→9
Absorption correction: multi-scan (Blessing, 1995)	*k* = −9→10
*T*_min_ = 0.958, *T*_max_ = 0.967	*l* = −22→22
15799 measured reflections	

### Refinement

**Table:**

Refinement on *F*^2^	Primary atom site location: structure-invariant direct methods
Least-squares matrix: full	Secondary atom site location: difference Fourier map
*R*[*F*^2^ > 2σ(*F*^2^)] = 0.075	Hydrogen site location: inferred from neighbouring sites
*wR*(*F*^2^) = 0.224	H atoms treated by a mixture of independent and constrained refinement
*S* = 1.05	*w* = 1/[σ^2^(*F*_o_^2^) + (0.0971*P*)^2^ + 0.2008*P*] where *P* = (*F*_o_^2^ + 2*F*_c_^2^)/3
2334 reflections	(Δ/σ)_max_ = 0.001
145 parameters	Δρ_max_ = 0.49 e Å^−^^3^
2 restraints	Δρ_min_ = −0.19 e Å^−^^3^

### Special details

**Table:**

Geometry. Bond distances, angles *etc*. have been calculated using the rounded fractional coordinates. All su's are estimated from the variances of the (full) variance-covariance matrix. The cell e.s.d.'s are taken into account in the estimation of distances, angles and torsion angles
Refinement. Refinement on *F*^2^ for ALL reflections except those flagged by the user for potential systematic errors. Weighted *R*-factors *wR* and all goodnesses of fit *S* are based on *F*^2^, conventional *R*-factors *R* are based on *F*, with *F* set to zero for negative *F*^2^. The observed criterion of *F*^2^ > σ(*F*^2^) is used only for calculating -*R*-factor-obs *etc*. and is not relevant to the choice of reflections for refinement. *R*-factors based on *F*^2^ are statistically about twice as large as those based on *F*, and *R*-factors based on ALL data will be even larger.

### Fractional atomic coordinates and isotropic or equivalent isotropic displacement parameters (Å^2^)

**Table:**

	*x*	*y*	*z*	*U*_iso_*/*U*_eq_	
O1	0.2324 (5)	0.4012 (5)	1.02649 (18)	0.1262 (18)	
N1	0.3982 (4)	0.4236 (4)	0.8222 (2)	0.0689 (14)	
C1	−0.1184 (6)	0.1831 (6)	0.9398 (3)	0.090 (2)	
C2	−0.2586 (7)	0.1067 (7)	0.9575 (4)	0.114 (3)	
C3	−0.2887 (7)	0.0999 (7)	1.0303 (4)	0.110 (3)	
C4	−0.1824 (7)	0.1686 (8)	1.0833 (3)	0.103 (3)	
C5	−0.0433 (7)	0.2458 (6)	1.0658 (3)	0.088 (2)	
C6	−0.0099 (6)	0.2528 (5)	0.9940 (2)	0.0722 (17)	
C7	0.1440 (6)	0.3359 (6)	0.9778 (3)	0.0828 (19)	
C8	0.1897 (5)	0.3369 (5)	0.9007 (2)	0.0749 (17)	
C9	0.3548 (5)	0.4217 (5)	0.8982 (2)	0.0772 (17)	
C10	0.5624 (6)	0.5009 (5)	0.8146 (3)	0.0850 (19)	
C11	0.6181 (8)	0.4545 (7)	0.7460 (4)	0.133 (3)	
C12	0.5461 (6)	0.6727 (6)	0.8219 (3)	0.109 (3)	
Cl1	0.40593 (14)	0.07597 (13)	0.77578 (6)	0.0828 (5)	
H1	−0.09700	0.18770	0.89130	0.1080*	
H1N	0.321 (5)	0.453 (5)	0.788 (2)	0.0990*	
H2	−0.33240	0.06020	0.92100	0.1370*	
H2N	0.402 (6)	0.332 (3)	0.803 (2)	0.0990*	
H3	−0.38260	0.04780	1.04250	0.1320*	
H4	−0.20350	0.16370	1.13180	0.1240*	
H5	0.02880	0.29350	1.10240	0.1060*	
H8A	0.10170	0.38750	0.86830	0.0900*	
H8B	0.19980	0.23160	0.88380	0.0900*	
H9A	0.34510	0.52680	0.91550	0.0930*	
H9B	0.44310	0.37050	0.93010	0.0930*	
H10	0.64530	0.46460	0.85460	0.1020*	
H11A	0.53580	0.48440	0.70610	0.1990*	
H11B	0.63260	0.34440	0.74560	0.1990*	
H11C	0.72290	0.50400	0.74080	0.1990*	
H12A	0.65050	0.72110	0.81500	0.1630*	
H12B	0.51800	0.69720	0.86970	0.1630*	
H12C	0.45930	0.70980	0.78540	0.1630*	

### Atomic displacement parameters (Å^2^)

**Table:**

	*U*^11^	*U*^22^	*U*^33^	*U*^12^	*U*^13^	*U*^23^
O1	0.151 (3)	0.161 (4)	0.065 (2)	−0.063 (3)	0.007 (2)	−0.016 (2)
N1	0.065 (2)	0.066 (2)	0.076 (3)	−0.0063 (19)	0.0105 (16)	0.0006 (18)
C1	0.093 (4)	0.099 (4)	0.083 (3)	−0.006 (3)	0.027 (3)	−0.010 (3)
C2	0.082 (4)	0.143 (6)	0.119 (5)	−0.017 (3)	0.022 (3)	−0.019 (4)
C3	0.087 (4)	0.138 (5)	0.113 (5)	0.003 (3)	0.039 (4)	0.015 (4)
C4	0.091 (4)	0.136 (5)	0.086 (4)	0.025 (4)	0.028 (3)	0.020 (3)
C5	0.094 (4)	0.102 (4)	0.069 (3)	0.013 (3)	0.012 (3)	0.003 (3)
C6	0.078 (3)	0.072 (3)	0.068 (3)	0.009 (2)	0.015 (2)	−0.002 (2)
C7	0.097 (4)	0.086 (3)	0.065 (3)	0.002 (3)	0.009 (2)	−0.003 (2)
C8	0.084 (3)	0.075 (3)	0.067 (3)	−0.007 (2)	0.015 (2)	−0.001 (2)
C9	0.081 (3)	0.078 (3)	0.072 (3)	−0.004 (2)	0.007 (2)	0.002 (2)
C10	0.069 (3)	0.069 (3)	0.118 (4)	−0.005 (2)	0.016 (3)	0.003 (3)
C11	0.127 (5)	0.131 (5)	0.151 (6)	−0.037 (4)	0.061 (4)	−0.027 (4)
C12	0.090 (4)	0.069 (3)	0.168 (6)	−0.015 (3)	0.021 (3)	0.002 (3)
Cl1	0.0792 (8)	0.0703 (8)	0.0974 (9)	0.0004 (6)	0.0050 (6)	−0.0080 (6)

### Geometric parameters (Å, º)

**Table:**

O1—C7	1.212 (6)	C1—H1	0.9300
N1—C9	1.483 (5)	C2—H2	0.9300
N1—C10	1.502 (6)	C3—H3	0.9300
N1—H1N	0.87 (4)	C4—H4	0.9300
N1—H2N	0.87 (3)	C5—H5	0.9300
C1—C2	1.380 (8)	C8—H8A	0.9700
C1—C6	1.379 (7)	C8—H8B	0.9700
C2—C3	1.392 (10)	C9—H9A	0.9700
C3—C4	1.352 (9)	C9—H9B	0.9700
C4—C5	1.375 (8)	C10—H10	0.9800
C5—C6	1.382 (7)	C11—H11A	0.9600
C6—C7	1.493 (7)	C11—H11B	0.9600
C7—C8	1.509 (7)	C11—H11C	0.9600
C8—C9	1.522 (6)	C12—H12A	0.9600
C10—C12	1.500 (7)	C12—H12B	0.9600
C10—C11	1.448 (9)	C12—H12C	0.9600
			
C9—N1—C10	113.9 (3)	C5—C4—H4	120.00
C10—N1—H1N	111 (3)	C4—C5—H5	120.00
C9—N1—H1N	117 (3)	C6—C5—H5	120.00
C9—N1—H2N	113 (2)	C7—C8—H8A	110.00
C10—N1—H2N	107 (3)	C7—C8—H8B	110.00
H1N—N1—H2N	92 (4)	C9—C8—H8A	110.00
C2—C1—C6	120.0 (5)	C9—C8—H8B	110.00
C1—C2—C3	119.5 (6)	H8A—C8—H8B	108.00
C2—C3—C4	120.4 (5)	N1—C9—H9A	110.00
C3—C4—C5	120.3 (5)	N1—C9—H9B	110.00
C4—C5—C6	120.4 (5)	C8—C9—H9A	110.00
C5—C6—C7	118.5 (4)	C8—C9—H9B	110.00
C1—C6—C7	122.1 (4)	H9A—C9—H9B	108.00
C1—C6—C5	119.4 (5)	N1—C10—H10	108.00
O1—C7—C6	120.1 (5)	C11—C10—H10	108.00
C6—C7—C8	119.7 (4)	C12—C10—H10	108.00
O1—C7—C8	120.2 (4)	C10—C11—H11A	109.00
C7—C8—C9	110.3 (3)	C10—C11—H11B	109.00
N1—C9—C8	110.0 (3)	C10—C11—H11C	109.00
N1—C10—C12	110.2 (4)	H11A—C11—H11B	110.00
C11—C10—C12	113.1 (5)	H11A—C11—H11C	109.00
N1—C10—C11	109.2 (4)	H11B—C11—H11C	109.00
C2—C1—H1	120.00	C10—C12—H12A	109.00
C6—C1—H1	120.00	C10—C12—H12B	110.00
C1—C2—H2	120.00	C10—C12—H12C	109.00
C3—C2—H2	120.00	H12A—C12—H12B	110.00
C2—C3—H3	120.00	H12A—C12—H12C	109.00
C4—C3—H3	120.00	H12B—C12—H12C	109.00
C3—C4—H4	120.00		
			
C10—N1—C9—C8	−178.2 (3)	C4—C5—C6—C1	0.7 (8)
C9—N1—C10—C11	161.9 (4)	C4—C5—C6—C7	−179.1 (5)
C9—N1—C10—C12	−73.3 (5)	C5—C6—C7—C8	176.1 (4)
C2—C1—C6—C7	179.6 (5)	C1—C6—C7—O1	176.9 (5)
C6—C1—C2—C3	−0.4 (8)	C1—C6—C7—C8	−3.7 (7)
C2—C1—C6—C5	−0.2 (8)	C5—C6—C7—O1	−3.3 (7)
C1—C2—C3—C4	0.6 (9)	O1—C7—C8—C9	2.1 (6)
C2—C3—C4—C5	−0.1 (10)	C6—C7—C8—C9	−177.3 (4)
C3—C4—C5—C6	−0.6 (9)	C7—C8—C9—N1	−179.5 (4)

### Hydrogen-bond geometry (Å, º)

**Table:**

*D*—H···*A*	*D*—H	H···*A*	*D*···*A*	*D*—H···*A*
N1—H1*N*···Cl1^i^	0.87 (4)	2.30 (4)	3.141 (4)	163 (4)
N1—H2*N*···Cl1	0.87 (3)	2.27 (3)	3.131 (4)	169 (4)
C10—H10···O1^ii^	0.98	2.56	3.284 (7)	130

Symmetry codes: (i) −*x*+1/2, *y*+1/2, −*z*+3/2; (ii) −*x*+1, −*y*+1, −*z*+2.
